# DNA Double-Strand Breaks Induced in Human Cells by Twelve Metallic Species: Quantitative Inter-Comparisons and Influence of the ATM Protein

**DOI:** 10.3390/biom11101462

**Published:** 2021-10-05

**Authors:** Muriel Viau, Laurène Sonzogni, Mélanie L. Ferlazzo, Elise Berthel, Sandrine Pereira, Larry Bodgi, Adeline Granzotto, Clément Devic, Béatrice Fervers, Laurent Charlet, Nicolas Foray

**Affiliations:** 1 Inserm, U1296 Unit, Radiation: Defense, Health, Environment, Centre Léon-Bérard, 69008 Lyon, France; muriel.viau@inserm.fr (M.V.); laurene.sonzogni@inserm.fr (L.S.); melanie.ferlazzo@inserm.fr (M.L.F.); elise.berthel@inserm.fr (E.B.); sandrine.pereira@inserm.fr (S.P.); larry.bodgi@inserm.fr (L.B.); adeline.granzotto@inserm.fr (A.G.); clement.devic@inserm.fr (C.D.); 2 Cancer & Environment Department, Centre Léon-Bérard, 69008 Lyon, France; beatrice.fervers@lyon.unicancer.fr; 3 ISTerre Team, University Grenoble Alpes, 38000 Grenoble, France; charlet38@gmail.com

**Keywords:** metal toxicity, metal carcinogenesis, DNA double-strand breaks, ATM

## Abstract

Despite a considerable amount of data, the molecular and cellular bases of the toxicity due to metal exposure remain unknown. Recent mechanistic models from radiobiology have emerged, pointing out that the radiation-induced nucleo-shuttling of the ATM protein (RIANS) initiates the recognition and the repair of DNA double-strand breaks (DSB) and the final response to genotoxic stress. In order to document the role of ATM-dependent DSB repair and signalling after metal exposure, we applied twelve different metal species representing nine elements (Al, Cu, Zn Ni, Pd, Cd, Pb, Cr, and Fe) to human skin, mammary, and brain cells. Our findings suggest that metals may directly or indirectly induce DSB at a rate that depends on the metal properties and concentration, and tissue type. At specific metal concentration ranges, the nucleo-shuttling of ATM can be delayed which impairs DSB recognition and repair and contributes to toxicity and carcinogenicity. Interestingly, as observed after low doses of ionizing radiation, some phenomena equivalent to the biological response observed at high metal concentrations may occur at lower concentrations. A general mechanistic model of the biological response to metal exposure based on the nucleo-shuttling of ATM is proposed to describe the metal-induced stress response and to define quantitative endpoints for toxicity and carcinogenicity.

## 1. Introduction

Metals are abundantly used and transformed by a number of industrial activities such as mining, metallurgy, production of fertilizers, paints, batteries, and more recently, high-tech products. Accidental, environmental occupational exposures to metals have increased our knowledge about their toxicity. This is notably the case for lead (Pb) with saturnism [1] and cadmium (Cd) with Itai-Itai disease [2,3]. Aluminum (Al) and iron (Fe) have been incriminated in Alzheimer’s disease [4]. Copper (Cu) and Fe have been cited for their potential link to Parkinson’s disease [5,6]. In addition to their potential toxicity, chronic exposure to metals may also increase cancer risk. For example, epidemiological studies of workers in chromium (Cr) production and occupational and environmental exposure to arsenic (As) and nickel (Ni) have documented the risk of lung and nasal cancers [7,8,9,10]. 

Metals, therefore, represent an actual issue of public health throughout two major clinical features: *toxicity* and *cancer risk*. However, despite a considerable amount of data, the molecular and cellular bases of metal-induced (MI) toxicity and/or carcinogenesis remain unknown. This statement is notably explained by the fact that exposure to metals is various and the mechanisms related to toxicity and carcinogenesis are complex.

Interestingly, the majority of studies about MI biological effects have focused on base damage (BD) and/or DNA single-strand breaks (SSB) while there is still no clear *quantified* link between these types of DNA damage and cell death (toxicity) or cell transformation (cancer) [1,2,11,12]. By contrast, studies about the biological effects of ionizing radiation, another toxic *and* carcinogenic agent, provide increasing evidence of a *causal and quantified* link between toxicity and *unrepaired* DNA double-strand breaks (DSB) on one hand, and genomic instability and *misrepaired* DSB on another hand [13,14]. However, unlike radiation-induced (RI) stress, MI stress was long considered to be too energetically low to produce DSB. However, Fenton-like reactions and genetic impairments in the DSB repair pathways may cause indirect production of DSB in response to metal exposure. This hypothesis has been documented both *in vivo* and *in vitro* by using different DSB assays [1,2,15,16,17,18,19,20,21,22]. 

In quiescent mammalian cells, the DSB recognition and repair is mainly ensured by the predominant non-homologous end-joining (NHEJ) pathway whose phosphorylation of variant H2AX histone proteins (γH2AX) is the earliest sensor [23]. Recently, the rate of RI nucleo-shuttling of the ATM protein (RIANS), a major actor of DSB repair and signalling, was found to be a reliable predictor of radiosensitivity and cellular toxicity [24,25,26,27,28,29,30]. The following mechanistic model was proposed: RI oxidization triggers the monomerization of the cytoplasmic ATM dimers, which allows the ATM monomers to diffuse in the nucleus. Once in the nucleus, the ATM monomers phosphorylate H2AX histones, which triggers the formation of nuclear γH2AX foci and DSB recognition and repair by NHEJ [24,29,30,31]. A delay in the RIANS may be caused by an overproduction of some ATM phosphorylation substrate proteins in the cytoplasm that sequestrate the ATM monomers. Consequently, less ATM monomers diffuse in the nucleus, and two scenarios can be evoked: either the DSB that are non-recognized by NHEJ remain unrepaired and participate in cell death and toxicity, or they are repaired by error-prone recombination-like pathways and participate as misrepaired DNA damage in cell transformation and cancer [24,29,30,31]. Here, we examined whether the nucleo-shuttling of the ATM protein is also relevant for describing the response to MI stress. Human cells from different genetic statuses and origin were exposed to 12 different metallic species (representing nine different metals) and were subjected to immunofluorescence with the RIANS biomarkers as endpoints. 

## 2. Materials and Methods

### 2.1. Cell Lines 

For the first steps of the study, untransformed human fibroblast cell lines were used for their genomic stability. Cells were routinely cultured at 37 °C in 5% CO_2_ humid conditions as monolayers with Dulbecco’s modified Eagle’s medium (DMEM) (Gibco-Invitrogen-France, Cergy-Pontoise, France), supplemented with 20% fetal calf serum, penicillin, and streptomycin. All the experiments were performed with cells in the plateau phase of growth (95–99% in G0/G1) to overcome any cell cycle effects. The radiobiological features of the radioresistant 149BR, 1BR3, and HF19 (ECACC, Public Health England, Salisbury, UK) and the radiosensitive 01HNG fibroblasts were published elsewhere [32,33]. The 01HNG cell lines belongs to the “COPERNIC” collection managed by our lab and approved by the regional Ethical Committee. Cell lines were declared under the numbers DC2008-585, DC2011-1437 and DC2021-3957 to the Ministry of Research. Radiobiological database was protected under the reference as IDDN.FR.001.510017.000.D.P.2014.000.10300.

It is noteworthy that cellular radioresistance is generally defined as a clonogenic cell survival fraction at 2 Gy higher than 50% [14,32,33]. In the next steps of the study, other cellular models were used, notably endothelial and brain cells. The human mammary endothelial HMEC cells (#A10565; Thermo Fisher Scientific, Waltham, MA, USA were routinely cultured as monolayers with RPMI medium (Thermo Fisher Scientific), supplemented with 20% fetal calf serum, penicillin, and streptomycin [34]. The human spinal cord (Hasp), cortex (Ha), and hippocampus (Hah) were purchased from ScienCell Research Laboratories (Carlsbad, CA, USA) and routinely cultured as monolayers with a specific culture medium provided by the manufacturer. 

### 2.2. Metals

All the indicated metal species tested in this study, which included Al^3+^, Fe^3+^, Cu^2+^, Zn^2+^, Ni^2+^, Pd^2+^, Cd^2+^, Pb^2+^, and CrO_4_^2−^ (as AlCl_3_, CuCl_2_, CuSO_4_, ZnCl_2_, NiCl_2_, PdCl_2_, CdCl_2_, Cd(CH_3_CO_2_)_2_, C_12_H_10_Cd_3_O_14_, Pb(NO_3_)_2_, Na_2_CrO_4_, and FeCl_3_), were purchased from (Sigma-Aldrich France, Saint-Quentin-Fallavier, France). Metals were diluted into the culture medium for 24 h. If cells were irradiated, the culture medium was renewed without metal. It is noteworthy that the concentrations of the metal species tested in this study are much higher than those of these culture mediums. The beginning of irradiation or the introduction of metal in the culture medium served as “zero” post-stress time.

### 2.3. Treatment with Zoledronate and Pravastatine (ZoPra)

The ZoPra treatment was applied as previously published [35]. Briefly, cells were incubated with 1 µM pravastatine (Sigma-Aldrich France) in phosphate buffered saline solution (PBS) for 24 h at 37 °C. Thereafter, 1 µM zoledronate (Sigma-Aldrich France) in PBS was added into the culture medium, and cells were incubated for 12 h at 37 °C. The culture medium was renewed immediately before irradiation.

### 2.4. Irradiations

An X-ray clinical irradiator (Philips orthovoltage; Philips, Amsterdam, The Netherlands) devoted to research was used to perform all the irradiations. The X-ray beam was produced from a tungsten anode, applying a voltage setting of 200 kV, an intensity of 20 mA, and using a filtration of 0.1 mm copper filter. The dose-rate was 1.234 Gy/min [32].

### 2.5. Immunofluorescence

The immunofluorescence protocol was described elsewhere [36,37]. Briefly, cells were fixed in paraformaldehyde for 10 min at room temperature and were permeabilized in 0.5% Triton X-100 solution for 5 min at 4 °C. Primary and secondary antibody incubations were performed for 40 and 20 min at 37 °C, respectively. The anti-*γH2AX^ser139^* antibody (#05636; Upstate Biotechnology-Euromedex, Mundolsheim, France) was used at 1:800. The monoclonal anti-mouse anti-*pATM^ser1981^* (#ab2888) from Abcam (Cambridge, UK) was used at 1:100. Incubations with anti-mouse fluorescein (FITC) and rhodamine (TRITC) secondary antibodies were performed at 1:100 at 37 °C for 20 min. Slides were mounted in 4’,6’ Diamidino-2-Phényl-indole (DAPI)-stained Vectashield (Abcys, Paris, France) for scoring micronuclei and mitoses and examined with an Olympus fluorescence microscope. DAPI staining also allowed for indirectly evaluating the yield of G_1_ cells (nuclei with homogeneous DAPI staining), S cells (nuclei showing numerous γH2AX foci), G_2_ cells (nuclei with heterogeneous DAPI staining), and metaphase (visible chromosomes). In certain conditions described in this study, some nuclei may appear with hundreds of foci: these cells, called “highly damaged cells” (HDC), are generally observed after a cancer-prone hyper-recombination process [34,38]. In this study, the number of HDC cells was considered as a molecular endpoint.

The foci scoring procedure applied here has received the certification agreement of a CE mark and ISO-13485 quality management system norms. Our foci scoring procedure also developed some features that are protected in the frame of the Soleau Envelop and patents (FR3017625 A1, FR3045071 A1, EP3108252 A1) [37]. More than 50 nuclei were analyzed per experiment and at least 3 independent replicates were performed for each condition. The Gaussian nature of the distribution of the number of foci per cell was systematically controlled for unirradiated conditions and data was assessed 24 h post-irradiation and routinely for the other indicated post-irradiation times. Foci scoring performed by eye was characterized by a relative error ranging between 3 and 7%, which depends on the number of foci per cell. Such relative errors were found incompressible (a foci scoring based on a number of nuclei that would be 2, 5, or 10 times higher does not reduce the foci scoring error significantly (data not shown)). In our lab, inter-experiments and inter-reader differences were not statistically significant. Similarly, there was no significant difference between eye scoring and computerized ImageJ scoring when microscopy objective magnification is ×100 [24]. 

### 2.6. Statistical Analysis

The immunofluorescence data were fitted to the so-called Bodgi’s formula that describes the kinetics of the appearance/disappearance of nuclear foci formed by some protein relocalizing after genotoxic stress [39]. This formula also serves as a coherence control to analyze the γH2AX, pATM, and MRE11 data. Statistical analysis was performed using Kaleidagraph v4 (Synergy Software, Reading, PA, USA).

## 3. Results

### 3.1. Unrepaired MI DSB Assessed with Different Endpoints

**Figure 1:**
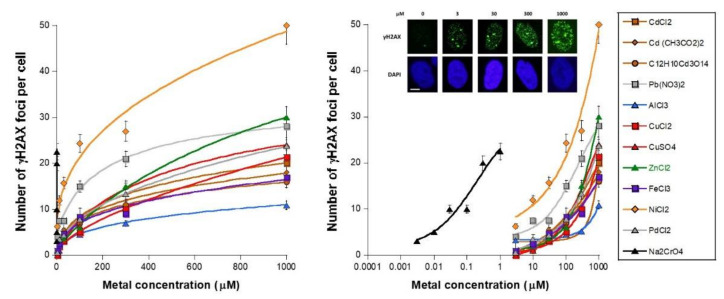
Unrepaired DSB after metal exposure. Number of γH2AX foci per cell in the human untransformed radioresistant 149BR cells after incubation for 24 h with the indicated concentration of metal species plotted either in a linear scale (left panel) or in a log scale (right panel). Each plot represents the mean ± standard error (SEM) of at least three replicates. Insert: Representative examples of γH2AX and DAPI-counterstained images obtained at the indicated concentration of CuCl_2_. The white bar represents 5 µm.

In our hands, the exposure of human cells to metal results in the production of metal-induced (MI) DSB whose occurrence may depend on the cellular model, the concentration, and the nature of the metallic species. Since the number of unrepaired RI DSB assessed at 24 h post-irradiation has been shown to be correlated with cellular death and therefore with toxicity [13,24], the number of MI DSB revealed by the nuclear γH2AX foci was assessed in human fibroblasts for 24 h after the introduction of metal in the culture medium. It is noteworthy that the shape of the γH2AX foci did not change with the nature of the metallic species (data not shown). The MI DSB response curves elicited similar sigmoidal functions of the metal concentration that appeared curvilinear when the X-axis is a linear scale of concentration. Three parts of the MI response curves were identified (Figure 1):

− a metal concentration range in which the number of γH2AX foci is not significant or does not increase significantly with metal concentration;
− a metal concentration range in which the number of γH2AX foci becomes significant and increases, generally linearly, with the metal concentration;
− a metal concentration range in which the number of γH2AX foci reaches its maximum in a pseudo-plateau.

All the mathematical features of the sigmoidal data fits are detailed in Table 1. 

It is noteworthy that similar numbers of γH2AX foci were obtained with other untransformed radioresistant fibroblast cell lines as those assessed in the 149BR cells, such as the HF19 and MRC5 cells (Figure S1).

In our conditions of culture, the average background level of non-transformed human fibroblasts was found to be lower than two γH2AX foci per cell [24,31], suggesting that a higher number of γH2AX foci per cell can reveal toxicity. The threshold metal concentration to reach more than two γH2AX foci per cell, called TMC_>2_, was deduced from experimental data. The metallic species tested were found to be characterized by a specific metal concentration threshold of some µM, with the notable exception of CrO_4_^2^^−^ whose corresponding TMC_>2_ was found in the nM range (Table 1). Our findings suggest that all the metallic species tested induce persistent/slowly repairable DSB at a specific rate (Figure 1).

**Figure 2:**
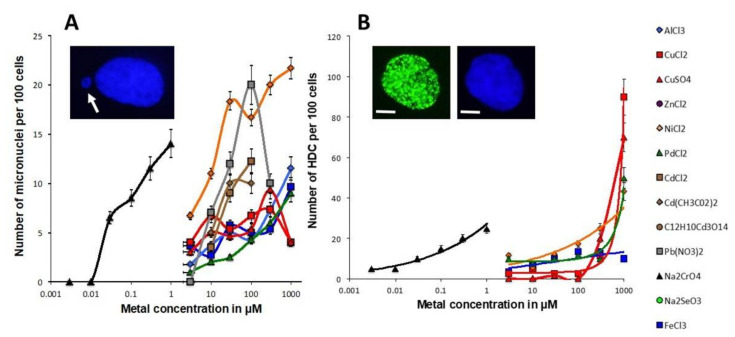
Residual micronuclei and highly damaged cells after metal exposure. (**A**) Number of micronuclei per 100 cells in the human untransformed radioresistant 149BR cells after incubation for 24 h with the indicated concentration of metal species. Each plot represents the mean ± standard error (SEM) of at least three replicates. Insert: Representative example of one micronucleus (white arrow). (**B**) Number of HDC per 100 cells in the human untransformed radioresistant 149BR cells after incubation for 24 h with the indicated concentration of metal species. Each plot represents the mean ± standard error (SEM) of at least three replicates. Insert: Representative example of one HDC stained with anti-γH2AX antibody and counterstained with DAPI. The white bar represents 5 µm.

In the same experimental conditions, we investigated other endpoints generally associated with unrepaired DSB such as the yields of micronuclei and highly damaged cells (HDC) (defined here as nuclei showing more than 15 γH2AX foci per cell) [34,38]. Micronuclei are considered to be the cytogenetic reflection of the unrepaired DSB propagated to the mitotic phase [40]. The number of MI micronuclei generally obeyed a pseudo-sigmoidal function of the metal concentration in agreement with the γH2AX data (Figure 2B). Moreover, the MI micronuclei curves of CrO_4_^2−^ again appeared shifted to the nM range. Interestingly, some metal species such as Pb^2+^ and Cu^2+^ showed a drastic decrease in the number of micronuclei at high metal concentrations. At this stage, we can evoke the possibility that the process of micronuclei was so rapid that a large number of them escape from cells (exocytosis phenomenon) during the immunofluorescence procedure, as previously reported [11]. 

By plotting the γH2AX data against the corresponding micronuclei data, a quantitative link appeared between the two endpoints: the higher the number of residual γH2AX foci, the higher the yield of micronuclei with metal-specific linear slopes (Figure S2A). The γH2AXfoci/micronuclei ratio was found to be 1.2 ± 0.3 (r = 0.8; *p* = 0.04). Pd^2+^ elicited the highest γH2AX/micronuclei ratio (Figure S2A). 

HDC (cells with more than 15 γH2AX foci) are generally observed after a cancer-prone hyper-recombination process [38] and/or at a very high level of oxidative stress [34]. The MI HDC curves were found to be very similar to the γH2AX ones, suggesting again a strong metal concentration-dependence (Figure 2B). However, it is noteworthy that the Cu species induced large numbers of HDC cells. The link between the number of γH2AX foci and that of HDC was also found to be linear for the great majority of metallic species tested (the higher the number of residual γH2AX foci, the higher the yield of HDC). The γH2AX foci /HDC ratio was found to be 1.0 ± 0.3 (r = 0.85; *p* = 0.04). Cu^2+^ and Ni^2+^ elicited the highest γH2AX/HDC ratio (Figure S2B).

Some authors have reported that low metal concentrations may stimulate cellular proliferation, which would favor mitosis induction [41,42,43,44,45]. To verify this hypothesis, mitoses were scored by using the DAPI counterstaining in the same experimental conditions as described above. For all the metallic species tested, we observed a yield of more than two mitoses per one hundred cells. This number decreased as far as the metal concentration increased, suggesting that high concentrations of metal trigger cell cycle arrest. For certain metallic species, at specific concentrations, the number of mitoses increased or described non-linear concentration-effects: the biological interpretation of these data and notably, the interplay between cell concentrations, clonogenicity, and influence on the cell cycle control remain to be elucidated (Figure S3). However, since the highest number of mitoses did not exceed 13 mitoses per 100 cells (obtained with CuCl_2_), it must be stressed that the great majority of quiescent cells remains in G0/G1 phase even after a metal exposure for 24 h for all the metallic species tested (Figure S3).

### 3.2. Influence of the Presence of Metal during the RI DSB Recognition and Repair Process

**Figure 3:**
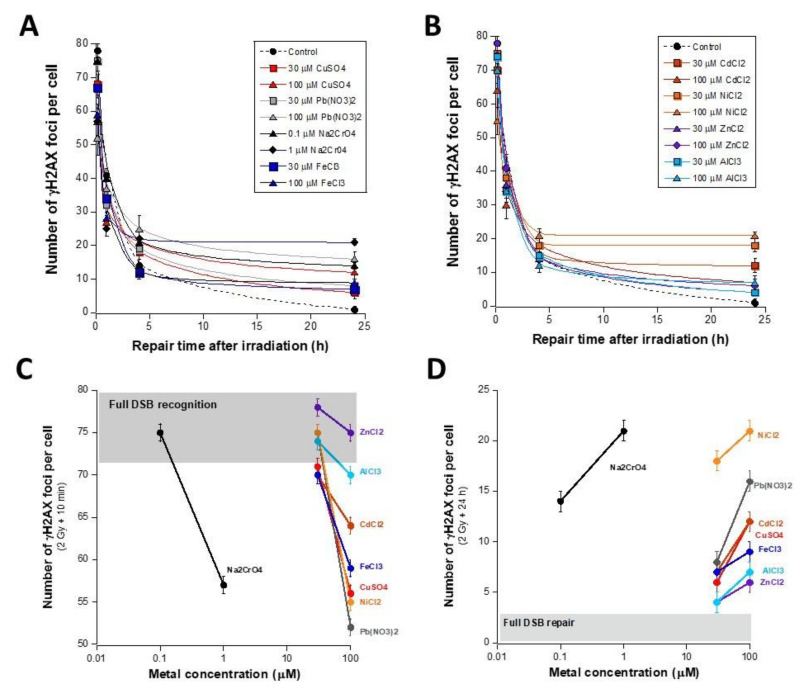
RI DSB repair kinetics after metal exposure. (**A**,**B**) The number of γH2AX foci as a function of repair time post-irradiation in the human untransformed radioresistant 149BR incubated for 24 h with the indicated concentration of metal species. Each plot represents the mean ± standard error (SEM) of at least three replicates. (**C**) The γH2AX data shown in panels (**A**,**B**) and obtained after 10 min post-irradiation were plotted against the metal concentration. The grey zone corresponds to the numerical values when all the RI DSB are recognized. A decrease in the number of γH2AX foci is interpreted as a defect in the DSB recognition. (**D**) The γH2AX data shown in panels A and B and obtained after 24 h post-irradiation were plotted against the metal concentration. The grey zone corresponds to the numerical values when all the RI DSB are unrepaired. An increase in the number of γH2AX foci is interpreted as a defect in the DSB repair.

The data described above showed that metal exposure induces a relatively low number of MI DSB at concentrations lower than 100 µM. Such data did not permit the verification of whether each metallic species inhibits recognition and/or repair of the DSB that it induces. Hence, to understand the influence of metal on the DSB recognition and repair steps, we used a physical agent to induce DSB without any chemical interaction with metal, the X-ray, as applied in previous studies [11,12]. The advantage of X-ray irradiation is that the RI DSB induction rate and the RI DSB repair kinetics of the cell lines tested are very well documented. Notably, in our conditions, a DSB induction rate of 37 ± 4 DSB per Gy per cell was found in human fibroblasts, in agreement with a number of reports [14]: at a dose of 2 Gy applied for 2 min, the number of RI DSB was much higher than the MI DSB. Here, cells were therefore exposed to metal for 24 h, then exposed to 2 Gy X-rays. After culture medium renewal, a post-irradiation repair time ranging from 10 min to 24 h was applied (Figure 3A,B). The resulting γH2AX foci kinetics elicited a shape of curves similar to that of cells irradiated without metal, to the notable exception of:

− the 10 min–1 h data points, that may reveal a low number of γH2AX foci, generally interpreted as a lack of DSB recognition by the NHEJ pathway [24,31] (Figure 3C);
− the 24 h data points, that may reveal a significant number of residual γH2AX foci, generally interpreted as a DSB repair defect by the NHEJ pathway [24,31] (Figure 3D);

The great majority of the resulting data suggests that the presence of metal may affect DSB recognition and/or repair. However, the quantitative impact on these two steps was found to be strongly dependent on the metal considered and its concentration. For example, it appeared that 100 µM Pb(NO_3_)_2_, 100 µM NiCl_2_, or 1 µM Na_2_CrO_4_ drastically impair the RI DSB recognition at 10 min post-irradiation, while 30 and 100 µM ZnCl_2_, 30 µM AlCl_3_, and 30 µM NiCl_2_ do not influence it (Figure 3C). By considering the DSB recognition after 100 µM metal exposure, the rank order of the metallic species tested was:

Pb(NO_3_)_2_ < NiCl_2_ < CuSO_4_ < FeCl_3_ < CdCl_2_ < AlCl_3_ < ZnCl_2_ (from the most impaired to the least). Na_2_CrO_4_ applied at 1 µM may be included in this order (between CuSO_4_ and FeCl_3_) by considering the number of non-recognized DSB.

With regard to the number of γH2AX foci obtained after 24 h post-irradiation, all the metallic species tested produced unrepaired MI DSB with the concentrations tested (Figure 3D). However, the rank order of the metallic species tested was (from the most defective repair to the least) very similar to that deduced from the DSB recognition data after 100 µM metal exposure: NiCl_2_ > Pb(NO_3_)_2_ > CuSO_4_ > FeCl_3_ > CdCl_2_ > AlCl_3_ > ZnCl_2_. Only the NiCl_2_ and Pb(NO_3_)_2_ data were found inverted in the two rank orders: NiCl_2_ produced more unrepaired DSB than Pb(NO_3_)_2_, while Pb(NO_3_)_2_ produced less unrecognized DSB than NiCl_2_. Na_2_CrO_4_ applied at 1 µM may be included between CuSO_4_ and FeCl_3_ by considering the number of unrepaired DSB.

Hence, in the presence of metal, RI DSB recognition data appears to be correlated to the RI DSB repair data: by plotting the number of γH2AX foci obtained after 24 h post-irradiation and the corresponding data obtained without irradiation, Figure S4 revealed strong RI DSB repair defects in agreement with the data shown in Figure 3A,B. Furthermore, a one-to-one correlation was found between the number of unrepaired DSB after metal exposure with and without irradiation (y = −3.68 + 1.15 x; r = 0.95; *p* < 0.02) (Figure S4). It is noteworthy that the non-nil intercept of about three γH2AX foci observed with X-ray treatment may correspond to the number of residual γH2AX foci generally assessed with the cell line tested after 2 Gy X-rays without metal exposure (the number of spontaneous γH2AX foci was not significant, data not shown) [24].

### 3.3. Influence of the Presence of Metal on the Nucleo-Shuttling of the ATM Protein

**Figure 4:**
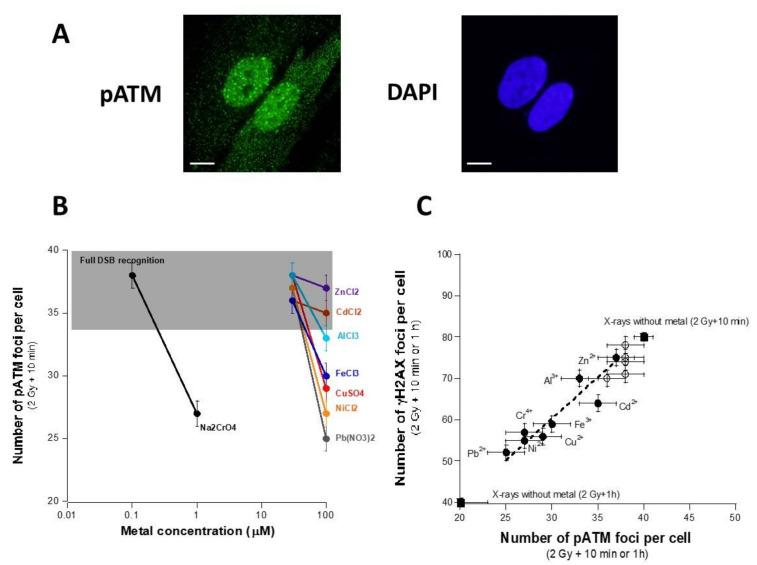
Delay of the ATM nucleo-shuttling after metal exposure. In the same conditions as described in Figure 3, anti-pATM immunofluorescence was applied to the radioresistant 149BR control cells to obtain pATM foci kinetics. (**A**) Representative example of images stained with *anti-pATM* antibody and counterstained with DAPI after 24 h exposure to 100 µM AlCl_3_, X-ray irradiation (2 Gy) followed by 10 min for repair. The white bar represents 10 µm. (**B**) The pATM data obtained after 10 min post-irradiation were plotted against the metal concentration. The grey zone corresponds to the numerical values when all the RI DSB are recognized. A decrease in the number of pATM foci is interpreted as a defect in the DSB recognition. Each plot represents the mean ± standard error (SEM) of at least three replicates. (**C**) The pATM data obtained after 100 mM metal exposure and 10 min (empty circles) or 1 h (black circles) post-irradiation shown in panel B were plotted against the corresponding γH2AX data shown in Figure 3. The black squares indicate the γH2AX and pATM data without metal exposure. Each plot represents the mean ± standard error (SEM) of at least three replicates. The dotted line represents the data fit with y = 2.0039x (r = 0.95) obtained from the 10 min data.

The data described above suggest that the presence of metal inhibits DSB recognition and/or repair via the ATM-dependent NHEJ pathway. Therefore, we examined the nuclear relocalization of the auto-phosphorylated form of the ATM protein, reflecting its kinase activity in the nucleus [31]. By applying *anti-pATM* immunofluorescence to cells exposed to metal and IR, the number of the nuclear pATM foci assessed 10 min after irradiation were found to be generally lower than those obtained with non-exposed cells, in good agreement with the γH2AX data (Figure 4A,B). Interestingly, when metal species were classified by their capacity of inhibiting DSB recognition, the rank order obtained with pATM and γH2AX data were found to be not significantly different (Figure 4C). A quantified correlation was also obtained between the two data groups with a γH2AX/pATM ratio of two, in full agreement with the RIANS model [28,31]. Again, these findings strongly suggest that metal exposure may impair the recognition of DSB by the ATM-dependent NHEJ pathway, which also impacts the quality of the DSB repair. Altogether, our findings can be interpreted as the result of a delay of the nucleo-shuttling of the ATM protein as proposed in a number of published papers [30]. 

The combination of zoledronate and pravastatin (ZoPra) was shown to increase the number of pATM foci and reduce the radiosensitivity associated with numerous genetic syndromes, suggesting that the active ATM forms present in the nucleus without ZoPra treatment are not sufficient to ensure a normal response to radiation [30,37,46,47]. While zoledronate is an aminobisphosphonate agent known for its action against osteoporosis, and pravastatin belongs to the statins family known for their anticholesterol properties, the ZoPra treatment was shown to inhibit both farnesylation and geranylgeranylation of the progerin and prelamin A proteins, which permits higher permeabilization of the nuclear membranes to protein trafficking [48]. Hence, the ZoPra treatment of cells exposed to genotoxic stress is supposed to amplify and accelerate the nucleoshuttling of the ATM protein. In fibroblasts exposed to AlCl_3_ or CuSO_4_, the number of early γH2AX and pATM foci were found systematically higher in ZoPra-treated cells than in untreated controls at 10 min and 1 h post-irradiation. However, the differences found were not significant (Figure S5). Altogether, these preliminary data encourage us to further investigate the action of ZoPra treatment or any other agent that would accelerate the ATM nucleoshuttling in cells exposed to metals (Figure S5).

### 3.4. Influence of the Presence of Metal on the Activity of the MRE11 Nuclease

Once in the nucleus, the ATM monomers were notably shown to phosphorylate the MRE11 protein, which inhibits its nuclease activity and triggers the formation of nuclear MRE11 foci [13,30]. A technical report revealed that the kinetics of appearance of the γH2AX foci do not overlap those of the MRE11 foci in the first hour post-irradiation, suggesting that ATM- and NHEJ-dependent phosphorylation of H2AX histones may be independent of the MRE11 activity [49]. It is also noteworthy that, in quiescent cells, the MRE11 nuclease is localized in the nucleus while the great majority of ATM molecules are cytoplasmic. Hence, the RIANS model suggests that the NHEJ and MRE11-dependent pathways compete in cells with different kinetics and that DSB revealed by γH2AX foci or by MRE11 foci may not have the same fate [30,50]. Interestingly, an early increase in the number of MRE11 foci revealing DNA strand break sites that are not managed by NHEJ has been correlated with the hyper-recombination process and cancer-proneness [13,30,50]. 

**Figure 5:**
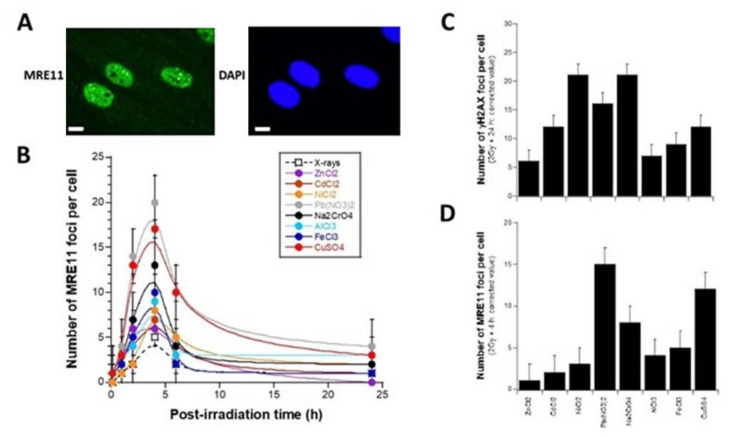
MRE11 foci after exposure of cells to metal. In the same conditions as described in Figure 3 (but only 100 µM metal was applied), anti-MRE11 immunofluorescence was applied to the radioresistant 149BR control cells. (**A**) Representative example of images stained with anti-MRE11 antibody and counterstained with DAPI after 24 h exposure to 100 µM AlCl_3_, X-ray irradiation (2 Gy) followed by 4 h for repair. The white bar represents 10 µm. (**B**) The MRE11 data was obtained after 100 µM metal incubation followed by 2 Gy X-rays and assessed at the indicated post-irradiation times. Each plot represents the mean ± standard error (SEM) of at least three replicates. (**C**) The number of residual γH2AX foci obtained after 100 µM metal as shown in Figure 3 but corrected with the γH2AX data without metal exposure was plotted at the indicated metallic species. (**D**) The number of the MRE11 foci obtained after 100 µM metal shown in Figure 5A but corrected with the MRE11 data without metal exposure was plotted at the indicated metallic species.

In order to examine the formation of MRE11 foci after a MI stress, fibroblasts were exposed to metal for 24 h and irradiated thereafter at 2 Gy X-rays. Anti-*MRE11* immunofluorescence was applied from 10 min to 24 h post-irradiation. While the 149BR cells that were not exposed to metal showed a slight increase in the number of MRE11 foci from 1 to 4 h post-irradiation, the exposures with the metallic species tested resulted in a significant increase in the number of MRE11 foci. The highest numbers of MRE11 foci values were generally reached at 4 h post-irradiation (Figure 5). If the metallic species tested were classified according to these maximal MRE11 foci values (from the highest to the lowest one), it would be as follows: Pb(NO_3_)_2_ > CuSO_4_ > Na_2_CrO_4_ > FeCl_3_ > AlCl_3_ > NiCl_2_ > CdCl_2_ > ZnCl_2_. Figure 5C,D reflects the differences between the number of residual γH2AX foci and the maximal number of MRE11 foci.

### 3.5. The Hypersensitivity Phenomenon Observed after Exposure to Low Metal Concentrations

**Figure 6:**
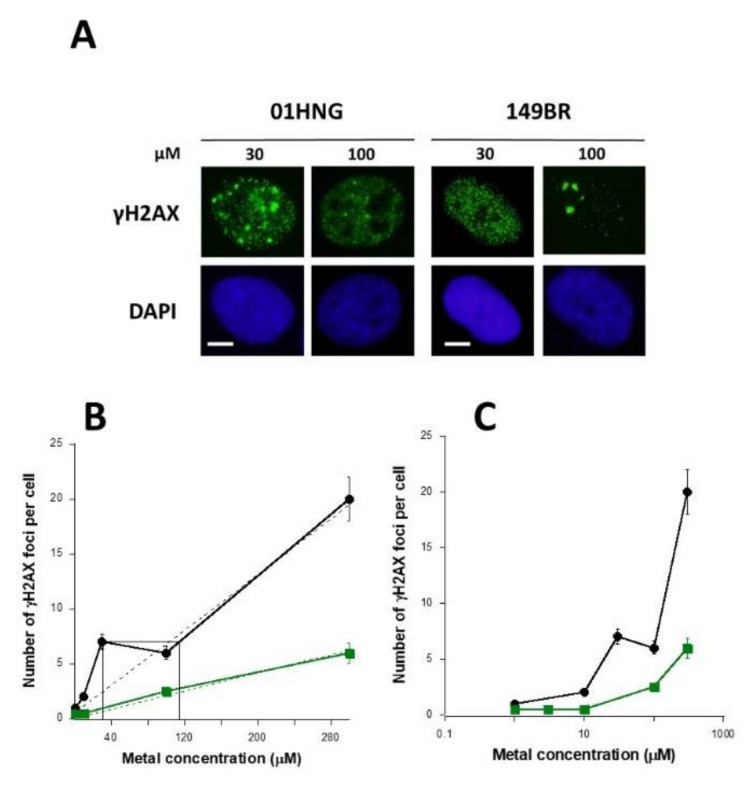
Evidence of an HRS-like phenomenon in cells exposed to metal. (**A**) Representative examples of images stained with anti-γH2AX antibody and counterstained with DAPI after 24 h exposure to 30 or 100 µM AlCl_3_ in the human radiosensitive 01HNG and the radioresistant 149BR skin fibroblast cells. (**B**,**C**) Number of γH2AX foci per cell in the 01HNG (black) and 149BR (green) cells after incubation for 24 h with the indicated concentration of AlCl_3_ presented in a linear (**B**) or a semi-log (**C**) scale. Each plot represents the mean ± standard error (SEM) of at least three replicates. Dotted lines represent linear data fits.

In radiation biology, the phenomenon called “hypersensitivity to low dose (HRS)” describes an excess of cell lethality, micronuclei, unrepaired DSB, or gene mutations in the [1 mGy − 800 mGy] dose range equivalent to the biology effect observed at a 5 to 10 times higher dose [31,51]. The HRS phenomenon is only observed in the cell lines in which the RIANS is delayed. In fact, in the frame of the RIANS model, this HRS phenomenon is supposed to be caused by a low number of ATM monomers (produced by low doses) in the cytoplasm that is not sufficient to recognize all the RI DSB after their diffusion into the nucleus. The HRS extent was shown to increase with the delay of RIANS [31]. If the RIANS model is relevant for MI DSB, some specific low metal concentrations may produce a similar phenomenon in cells with delayed nucleo-shuttling of the ATM protein. Therefore, we examined the occurrence of an HRS-like phenomenon after exposure to 30 µM AlCl_3_ in the 01HNG fibroblasts that show a significant delay in the RIANS [24]. AlCl_3_ was chosen because of its societal interest. Interestingly, in this cell line, the number of γH2AX foci was found to be equivalent to that expected at 115 µM AlCl_3_ (i.e., at about a 3.8 times higher concentration) (Figure 6A,B). This non-linear phenomenon was not observed with the radioresistant skin fibroblast 149BR cell line that shows a fast RIANS [24]. The HRS-like phenomenon may also be visible in a semi-log scale (Figure 6C). It is noteworthy that similar conclusions were reached with two other couples of radioresistant/radiosensitive fibroblast cell lines and also in the human endothelial HMEC cell lines (data not shown).

### 3.6. The Adaptive Response Observed after Exposure to Low Metal Concentrations

In parallel with the HRS phenomenon, another low radiation dose-specific phenomenon has recently been explained by the RIANS model: the adaptive response. Experimentally based on the succession of a low (d) and a high dose (D) separated by a certain period of time (Δt), the adaptive response is a phenomenon in which d + Δt + D provides a lower radiobiological effect than D alone [52,53,54]. In the frame of the RIANS model, in cells with delayed RIANS, the adaptive response occurs if the low dose, d, produces ATM monomers that accumulate in the nucleus without inducing DSB. If Δt is not too long, these ATM monomers will participate in the recognition and repair of DSB induced by D, in addition to the ATM monomers directly induced by D. Hence, the number of unrepaired DSB will be lower after d + Δt + D than after D applied alone. Such a phenomenon may not occur in cells without a delayed RIANS since the number of ATM monomers provided by D alone is sufficient to recognize all the DSB [52,53,54]. 

**Figure 7:**
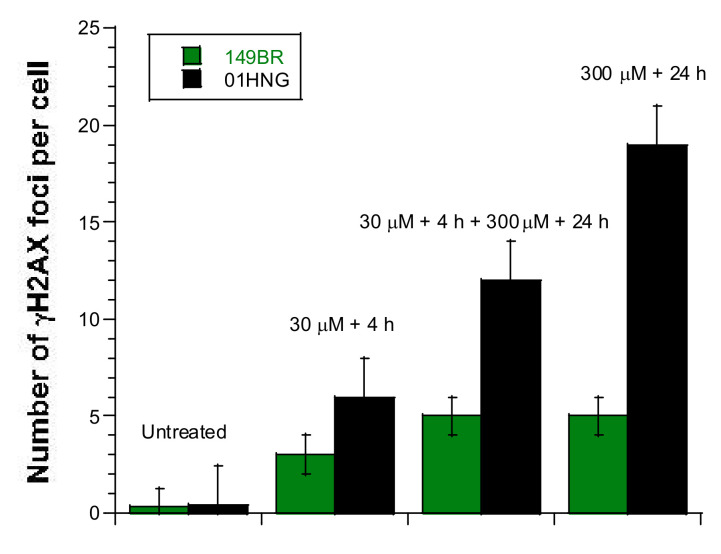
Evidence of an adaptive response-like phenomenon in cells exposed to metal. Number of γH2AX foci per cell in the 01HNG (black) and 149BR (green) cells after incubation with the indicated concentrations of AlCl_3_. Each bar represents the mean ± standard error (SEM) of at least three replicates.

In order to examine whether cells with delayed RIANS show an adaptive response-like phenomenon with metal exposure consisting in a succession of two concentrations c and C separated by a certain period of time Δt (i.e., c + Δt + C), we incubated cells with 30 µM AlCl_3_ for 4 h then with 300 µM AlCl_3_ for 24 h and the number of residual γH2AX foci per cell in the human radiosensitive 01HNG and the radioresistant 149BR fibroblast cells was assessed (Figure 7). AlCl_3_ was chosen because of its societal interest. Interestingly, while the radioresistant 149BR cells showed the same number of γH2AX foci after 30 µM + 4 h + 300 µM + 24 h and after 300 µM + 24 h, the 01HNG cells with delayed RIANS elicited significantly less γH2AX foci after 30 µM + 4 h + 300 µM + 24 h than after 300 µM + 24 h (*p* < 0.03), suggesting an adaptive response-like phenomenon (Figure 7). Similar conclusions were reached with pATM data (data not shown).

### 3.7. Cell-Type Dependence in the Response to Metal Exposure 

**Figure 8:**
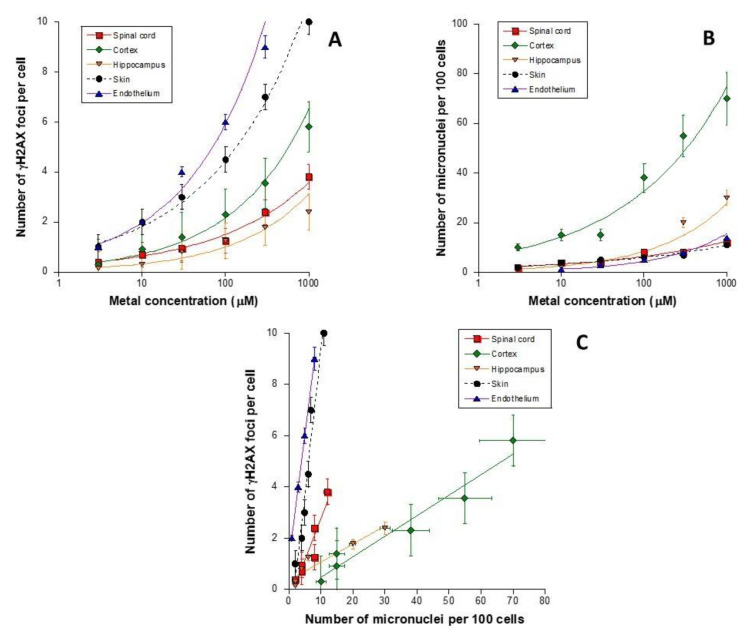
Evidence of a tissue-dependence in the response to metal exposure. (**A**,**B**). The number of γH2AX foci per cell (**A**) and micronuclei per 100 cells (**B**) in the human Hasp, Ha, Hah brain cells and the skin fibroblast 149BR cells after incubation for 24 h with the indicated concentration of AlCl_3_. Each plot represents the mean ± standard error (SEM) of at least three replicates. (**C**) The number of γH2AX foci shown in panel A was plotted against the corresponding number of micronuclei shown in panel B. Solid lines indicate the result of data fitting to a linear function.

All the above data were obtained with human skin fibroblasts. It is noteworthy that the RIANS model has been shown to be relevant for any other mammalian tissue [30]. The MI stress may also be strongly dependent on the nature of the tissue. Since the brain and endothelial cells may be concerned directly by metal exposure, the molecular response to MI stress of human brain cells was examined. To this aim, we assessed the number of γH2AX foci and micronuclei remaining after metal exposure for 24 h in three human non-transformed astrocyte cell lines and one endothelial cell line. These brain cell lines were provided from the cortex (Ha), the hippocampus (Hah), and the spinal cord (Hasp) from the same donor and were shown to elicit different delays of RIANS [55]. The 149BR fibroblast cell line served as a control (Figure 1). AlCl_3_ was chosen because of its societal interest. Similar to the data described in Figure 1, the number of residual γH2AX foci obeyed a pseudo-sigmoidal shape when metal concentration was plotted in a log scale. Particularly, the number of γH2AX foci in cortex Ha astrocytes were found higher than that in the spinal cord Hasp and the hippocampus Hah astrocytes (Figure 8A). Again, when micronuclei yields were considered as endpoints, the cortex astrocyte Ha cell line elicited much more micronuclei than the other two brain cell lines tested (Figure 8B). 

In order to better understand these findings, we plotted the number of residual γH2AX foci per cell against the corresponding number of micronuclei per 100 cells (Figure 8): while the skin fibroblast cell line described a linear γH2AX/micronuclei slope of about 1 (Figure 2), the slope value became 0.3 for the spinal cord astrocytes (similar to the slope obtained with X-rays) and 0.07 for the other two brain cell lines (Figure 8C), suggesting that 3 and 14 times more micronuclei are observed for the same number of γH2AX foci, respectively. The HMEC cells elicited similar characteristics as those assessed in the control fibroblasts (Figure 8). Similar conclusions were reached with pATM foci (data not shown). 

## 4. Discussion

### 4.1. The Need for a Consensual Scale to Account for Toxicity and Carcinogenicity of Metals

Due to their intense use in industry and their potential toxicity and carcinogenicity, metals, like any other chemical agents, are submitted to specific international and national rules in order to regulate their use and transport. Particularly, the material safety data sheets (MSDS) usefully inform workers and users of the main dangers that they represent. However, the MSDS content can differ drastically from one country to another. Faced with this diversity, international authorities endeavored to get consensual decisions with the Globally Harmonized System (GHS). Unfortunately, there is still no simple correspondence between all the categories proposed by the different safety agencies. GHS *categories* do not correspond to the *groups* defined by the International Agency for Research on Cancer (IARC); for example, a chemical classified as IARC Group 2B (*possibly carcinogen*) could be found in the GHS category 1B or category 2 (*presumed or suspected carcinogen*). All these statements demonstrate the need for a *quantitative and objective* scale to account for toxic and cancer risks, but also the necessity of examining a large panel of metal species with the same endpoints.

In addition to these statements, it must be stressed that like with some other chemical agents, exposure to metals likely leads to heterogeneous stress distribution in cells. Moreover, some metals may produce much higher intracellular concentrations than others. Hence, together with a *quantitative and objective* scale to account for toxic and cancer risks, dose-effect curves with a large range of metal concentrations are needed to document the biological effects of any metal exposure. Such quantification should also take into account the role of the specific action of anions for a given metal species. Here, even if this aspect was not the major scope of the study, the differences observed between CdCl_2_ and Cd(CH_3_CO_2_)_2_ are very representative of intra-species differences. 

Lastly, while the production of one RI DSB requires more than 100 eV per nm^3^ (this energy is reached at doses higher than 25 mGy, i.e., equivalent to that delivered during a standard chest CT scan exam) [14], exposure to metals was long considered to be too energetically low to directly produce numerous DSB [56]. However, the present study pointed out that metal exposure can result in the significant production of DSB in a non-linearly concentration-dependent manner. In the present study, we did not investigate the way by which MI DNA damage is induced. A number of biochemical models have been proposed to this aim and they suggest either direct or indirect formation of DNA damage produced by the presence of metal [16,57,58]. The relevance of the RIANS model applied to MI stress does not depend on the fact that MI DSB are directly or indirectly produced. However, the MI DNA damage production likely appears to be strongly dependent on the nature of the metallic species considered, and the biochemical and molecular features of the production of MI DSB need to be investigated further.

### 4.2. Unrepaired DSB as a Unit to Account for Toxicity of Metals?

According to the literature, the natural parameters for reflecting RI toxicity may be the number of unrepairable DNA damage and chromosome breaks [59]. However, in the case of metal exposure, our findings suggest two different categories of endpoints to account for MI toxicity: those defined in the presence of metal but after a well-characterized exposure to X-rays, and those defined in the presence of metal without exposure to X-rays. In the first category, the number of pATM foci assessed 10 min post-irradiation and the number of γH2AX foci assessed 24 h post-irradiation has been shown to be the best endpoints to predict radiosensitivity (i.e., RI toxicity) [13,24]. However, 2 Gy X-rays produce much more ATM monomers than the highest metal concentrations tested. Hence, even if X-ray irradiation is an interesting tool to study the molecular influence of metal in the DSB recognition and repair process, the endpoints requiring exposure to both X-rays and metal cannot easily be used to account for MI toxicity, in practice.

By contrast, by considering that the average background assessed in human fibroblasts does not exceed two spontaneous γH2AX foci per cell, the threshold metal concentration to reach more than two γH2AX foci per cell (TMC_>2_) has been defined (Table 1) and served as an endpoint of MI toxicity. However, the TMC_>2_ parameter may not include the HRS-like phenomenon observed and may be strongly dependent on the individual response to genotoxic stress. Since eight unrepaired RI DSB were shown to correspond to 100% RI cell lethality [13], the threshold metal concentration to reach more than eight γH2AX foci per cell (TMC_>8_) was also investigated (Table 2). Interestingly, TMC_>8_, better than TMC_>2_, appeared to be correlated to the number of γH2AX foci assessed 24 h post-irradiation and the number of γH2AX foci or pATM assessed 10 min post-irradiation (Figure S6). However, again, TMC_>8_, may reflect a range of MI toxicity that is rarely reached, even after accidental exposure. Hence, further experiments are needed to document better the interest and the validity domain of both TMC parameters.

In addition to the precited molecular endpoints, it is noteworthy that the RIANS model is also based on the ATM-metal complex affinity and stability. Some chemical features may reflect these features well. This is notably the case of the Misono softness parameter [60] that quantifies the soft character of the metal ions and their ability to form covalent bonds, notably with the ATM reactive base sites, according to the hard and soft acid and base principle [61] (i.e., corresponding to an increasing stability of the ATM-metal complexes) (Table 2). Interestingly, a quantitative correlation was found between the Misono softness parameter, the TMC_>8_, and the number of γH2AX foci assessed 24 h post-irradiation (Figure S6), supporting the importance of the chemical stability of the ATM-metal complexes in the nucleo-shuttling of the ATM protein: *the more stable the ATM-metal complexes, the larger the delay of the nucleo-shuttling of the ATM protein, the higher the toxicity of the metal.*

**Figure 9:**
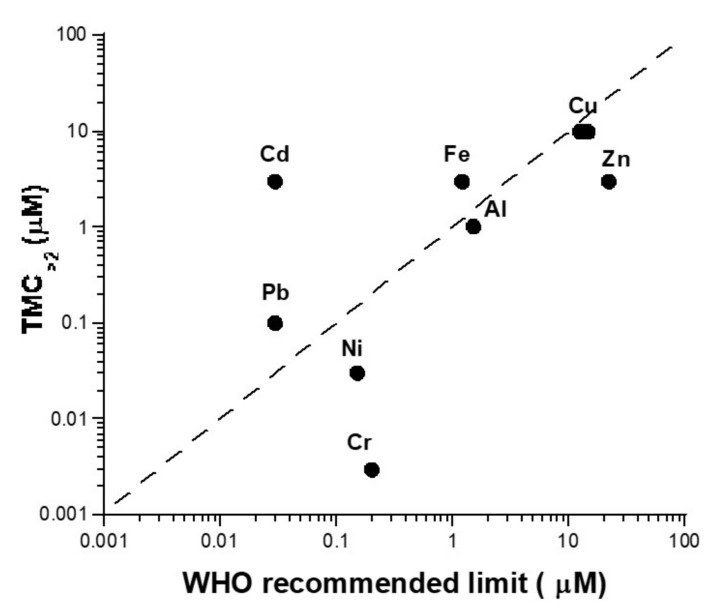
Comparison between TMC > 2 metal concentration limits in tap water. The TMC > 2 values shown in Table 1 and Table 2 were plotted against the metal concentration limits in tap water recommended by the WHO (Table 2). The dotted line corresponds to a one-to-one correlation.

How to test the biological relevance of the above parameters to reflect toxicity? By considering normal (radioresistant) human cells, and for each metallic species tested here, the TMC_>2_ was compared with the maximal concentrations of metal recommended in tap water by the World Health Organization (WHO) [62] (Table 2, Figure 9). With the notable exception of Cd and Cr, the TMC_>2_ values were found to be in acceptable agreement with the recommended concentration limits (i.e., six metal elements tested among eight). By contrast, TMC_>2_ values significantly underestimated the WHO recommendation for Cd, while it was the inverse for Cr (Figure 9). Further investigations are needed to document better the link between TMC and the metal concentration limits.

### 4.3. Misrepaired DSB as a Unit to Account for Carcinogenicity of Metals?

Likely because the intrinsic mechanisms of carcinogenesis are still unknown, one of the most important challenges of genotoxicology is to define reliable and very specific parameters for quantifying carcinogenic risk *separately from the toxicity risk* [50]; metal biology is not an exception. 

It is noteworthy that the ATM-dependent phosphorylation of H2AX (i.e., the formation of γH2AX foci) reflects the DSB recognized specifically by the NHEJ pathway. If the ATM nucleoshuttling is delayed, DSB may not be recognized by NHEJ but by the error-prone recombination-like pathway. Interestingly, in the particular case of cancer-prone syndromes, the error-prone recombination-like process may be out of control: this is the *hyper-recombination* phenomenon. The hyper-recombination phenomenon is responsible for the production of additional DSB that may not be revealed by γH2AX foci [50]. Since unrepaired DSB may lead to the formation of micronuclei indifferently of the DSB repair and signaling pathway, hyper-recombination may contribute to an increase of the number micronuclei faster than the number of γH2AX foci. Hence, the γH2AX foci/micronuclei ratio may be a promising endpoint for cancer-proneness while the number of residual γH2AX foci may be more specific to toxicity [50]. 

As developed in Section 3.5, in the frame of the RIANS model, abnormal MRE11 activity and abnormal MRE11 foci kinetics appear to be correlated to the hyper-recombination phenomenon and cancer proneness, but not necessarily to toxicity [13,30,50]. By plotting the Misono softness parameter against the number of γH2AX foci assessed 24 h post-irradiation (Figure S7A) and against the number of MRE11 foci assessed 4 h post-irradiation (Figure S7B), a certain linearity appears with the γH2AX data, but not with the MRE11 data (Figure S7), suggesting that the ATM-metal complex affinity and stability reflected by the Misono softness parameter may influence the γH2AX-dependent pathway but not the MRE11-dependent pathway. However, again, considering the relatively low number of MI DSB after realistic exposure to metal, the parameters based on the combination of RI and MI stress cannot be easily applied, in practice. Further investigations will be necessary to identify more specific endpoints to account for metal carcinogenicity. 

### 4.4. Toward a Unified Model for Understanding the Response to Metals?

**Figure 10:**
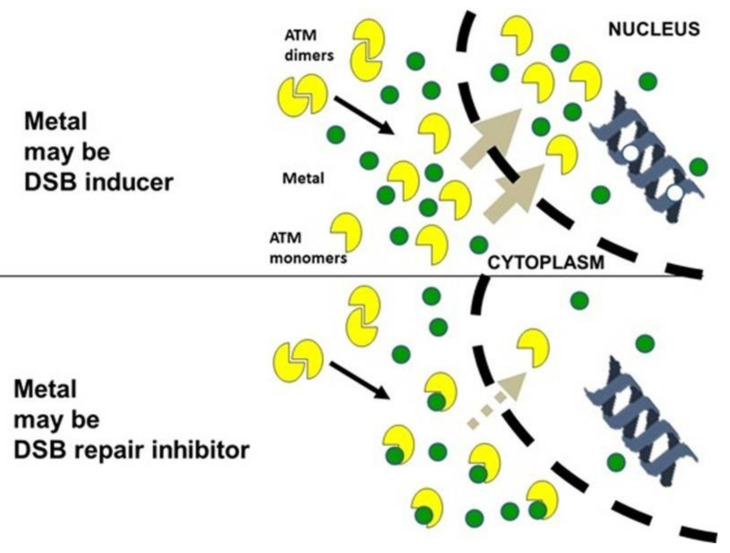
Mechanistic model of metal action. As detailed in the discussion, the presence of metal species may contribute to monomerizing ATM and/or inducing DSB. If the nucleo-shuttling of ATM is delayed, DSB will be not recognized by the NHEJ pathway. There are two possible consequences: either DSB are unrepaired, which triggers cell death and toxicity, or DSB are misrepaired by an alternative DSB repair pathway, which triggers genomic instability and carcinogenicity.

Our findings showed a coherent and quantitative link between γH2AX foci, pATM foci, MRE11 foci, micronuclei, and HDC cells for all the metallic species tested. Data are in agreement with a delay of the ATM nucleoshuttling that would occur at high concentrations depending on the metal. The HRS-like, the adaptive response phenomena, and the tissue-dependence of the response of metal exposure were also described and predicted by the RIANS model. Hence, a general mechanistic model can be proposed (Figure 10): metals that enter into cells can create, via direct or indirect Fenton-like reactions, an oxidative stress that induces ATM monomerization in the cytoplasm and DSB in the nucleus (similar to ionizing radiation and hydrogen peroxide [63]). The rates of ATM monomerization and of the formation of DSB are strongly dependent on cell type and metal species. At a certain threshold concentration of metal, metals form with ATM some complexes that delay the nucleo-shuttling of ATM. Such a delay prevents the full recognition and repair of MI DSB via NHEJ, which leads to toxicity and/or carcinogenicity. At low concentrations of metals, if the rate of ATM monomerization is too low, an HRS-like phenomenon may occur. Lastly, in cells with a large cytoplasm, the mean free path of ATM may be longer, which leads to a significant delay in the nucleoshuttling of ATM and influences the response to metal exposure (Figure 10). 

## 5. Conclusions

Our findings obtained with different assays and endpoints (γH2AX, pATM, MRE11, micronuclei) and with several metallic species suggest that exposure to metal leads to the production of DSB, whether direct or indirect, and the activation of the ATM protein kinase. Like after exposure to ionizing radiation, exposure to metal is consistent with a model based on the production of ATM monomers in the cytoplasm that diffuse into the nucleus at a specific rate and extent depending on the metallic species and on their concentration. At high metal concentrations, some ATM-metal complexes prevent the diffusion of ATM monomers in the nucleus, which impairs the DSB recognition and repair, leading to toxicity and/or carcinogenicity. Some specific biomarkers are proposed to better evaluate the risks associated with exposure to metal. 

While metal nanoparticles are currently used as theranostic agents for cancer radiotherapy, there is some evidence that they may also result in producing DSB that may participate in the response of treated tumors or exposed healthy tissues [64]. Further experiments will be therefore needed to link the data involving nanoparticles to those involving metal salts.

## 6. Patents

WO2017029450—Individual method predictive of the DNA-breaking genotoxic effects of chemical or biochemical agents.

## Figures and Tables

**Table 1:** Metal species characteristics and data fit parameters.

Metal Species	TMC_>2_ $$$$(µM)	Sigmoidal* Data Fit Parameters				
		m1	m2	m3	m4	r
Na_2_CrO_4_	0.003	30	1.71	1.90 × 10^−1^	0.67	0.97
NiCl_2_	0.03	3380	4.49	2.77 × 10^7^	0.42	0.98
Pb(NO_3_)_2_	0.1	35	3.69	2.31 × 10^2^	0.80	0.99
AlCl_3_	1	36	0.29	5.75 × 10^3^	0.48	0.99
FeCl_3_	3	735	0	6.53 × 10^7^	0.33	0.98
ZnCl_2_	3	72	1.00	1.50 × 10^3^	0.89	0.99
CdCl_2_	3	58	0	3.54 × 10^3^	0.46	0.99
Cd(CH_3_CO_2_)_2_	3	603	0	2.29 × 10^8^	0.26	0.99
C_12_H_10_Cd_3_O_14_	10	80	0	5.25 × 10^4^	0.28	0.99
PdCl_2_	10	1045	0	2.94 × 10^6^	0.47	0.99
CuSO_4_	10	43	0	7.07 × 10^2^	0.75	0.99
CuCl_2_	10	1501	0.13	8.84 × 10^5^	0.62	0.99

* The applied sigmoidal function tested for data fitting was y = m1 + (m2 − m1)/(1 + (x/m3) ^m4^). r is the correlation coefficient.

**Table 2:** Major biochemical and radiobiological values for the metallic species tested.

Metallic Species	Misono Softness Parameter	TMC > 2$$$$ (µM)	TMC > 8$$$$ (µM)	γH2AX Foci at 24 h ^2^	MRE11 Foci at 4 h ^3^	WHO Limits in Tap Water (µM)
Na_2_CrO_4_	na ^1^	0.003	0.06	21 (1 µM)	8 (1 µM)	0.2
NiCl_2_	2.82	0.03	9	21	3	0.15
Pb(NO_3_)_2_	3.58	0.1	30	16	15	0.03
AlCl_3_	1.6	1	1000	7	4	1.5
FeCl_3_	3.09	3	300	9	5	1.2
ZnCl_2_	2.34	3	700	6	1	22
CdCl_2_	3.04	3	100	12	2	0.03
Cd(CH_3_CO_2_)_2_	3.04	3	100	na ^1^	na ^1^	na ^1^
C_12_H_10_Cd_3_O_14_	3.04	10	100	na ^1^	na ^1^	na ^1^
PdCl_2_	na ^1^	10	100	na ^1^	na ^1^	na ^1^
CuCl_2_	2.89	10	800			14.9
CuSO_4_	2.89	10	100	12	12	12.5

^1^ Non available; ^2^ Number of γH2AX foci assessed 24 h post-irradiation and exposed to 100 µM with the notable exception of Na_2_CrO_4_ (data shown in Figure 3A,B and Figure 5C) corrected by the number of foci without metal concentration (irradiation only); ^3^ Number of MRE11 foci assessed 4 h post-irradiation and exposure to 100 µM with the notable exception of Na_2_CrO_4_ (data shown in Figure 5B,D) corrected by the number of foci without metal concentration (irradiation only).

## Data Availability

The data presented here are either present in a deposed database (see materials and methods) or will be made available on reasonable request.

## Supplementary Materials

The following are available online at https://www.mdpi.com/article/10.3390/biom11101462/s1, Figure S1: Mitoses and metal exposure; Figure S2: Effect of ZoPra treatment and exposure to metal; Figure S3: Correlation between γH2AX data obtained after 24 h exposure to metal followed by irradiation and γH2AX data obtained without exposure to metal; Figure S4: Correlations between TMC, number of γH2AX foci, and Misono softness parameters.

## References

[1] IARC (2006). Inorganic and organic lead compounds. IARC Monogr. Eval. Carcinog. Risks Hum..

[2] IARC (1993). Beryllium, cadmium, mercury, and exposures in the glass manufacturing industry. Working Group views and expert opinions, Lyon, 9–16 February 1993. IARC Monogr. Eval. Carcinog. Risks Hum.

[3] Bertin G., Averbeck D. (2006). Cadmium: Cellular effects, modifications of biomolecules, modulation of DNA repair and genotoxic consequences (a review). Biochimie.

[4] Drago D., Bolognin S., Zatta P. (2008). Role of metal ions in the abeta oligomerization in Alzheimer’s disease and in other neurological disorders. Curr. Alzheimer Res..

[5] Boll M.C., Alcaraz-Zubeldia M., Montes S., Rios C. (2008). Free copper, ferroxidase and SOD1 activities, lipid peroxidation and NO(x) content in the CSF. A different marker profile in four neurodegenerative diseases. Neurochem. Res..

[6] Charlet N., Chapron Y., Faller P., Kirsch R., Stone A.T., Baveye P.C. (2012). Neurodegenerative diseases and exposure to the environmental metals Mn, Pb, and Hg. Coord. Chem. Rev..

[7] Smith A.H., Goycolea M., Haque R., Biggs M.L. (1998). Marked increase in bladder and lung cancer mortality in a region of Northern Chile due to arsenic in drinking water. Am. J. Epidemiol..

[8] Smith A.H., Hopenhayn-Rich C., Bates M.N., Goeden H.M., Hertz-Picciotto I., Duggan H.M., Wood R., Kosnett M.J., Smith M.T. (1992). Cancer risks from arsenic in drinking water. Environ. Health Perspect..

[9] Smith A.H., Marshall G., Yuan Y., Ferreccio C., Liaw J., von Ehrenstein O., Steinmaus C., Bates M.N., Selvin S. (2006). Increased mortality from lung cancer and bronchiectasis in young adults after exposure to arsenic in utero and in early childhood. Environ. Health Perspect..

[10] Wild P., Bourgkard E., Paris C. (2009). Lung cancer and exposure to metals: The epidemiological evidence. Cancer Epidemiol..

[11] Gastaldo J., Viau M., Bencokova Z., Joubert A., Charvet A.M., Balosso J., Foray N. (2007). Lead contamination results in late and slowly repairable DNA double-strand breaks and impacts upon the ATM-dependent signaling pathways. Toxicol. Lett..

[12] Viau M., Gastaldo J., Bencokova Z., Joubert A., Foray N. (2008). Cadmium inhibits non-homologous end-joining and over-activates the MRE11-dependent repair pathway. Mutat. Res..

[13] Joubert A., Zimmerman K.M., Bencokova Z., Gastaldo J., Rénier W., Chavaudra N., Favaudon V., Arlett C., Foray N. (2008). DNA double-strand break repair defects in syndromes associated with acute radiation response: At least two different assays to predict intrinsic radiosensitivity?. Int. J. Radiat. Biol..

[14] Foray N., Bourguignon M., Hamada N. (2016). Individual response to ionizing radiation. Mutat. Res. Rev..

[15] Morales M.E., Derbes R.S., Ade C.M., Ortego J.C., Stark J., Deininger P.L., Roy-Engel A.M. (2016). Heavy Metal Exposure Influences Double Strand Break DNA Repair Outcomes. PLoS ONE.

[16] Rittich B., Spanova A., Falk M., Benes M.J., Hruby M. (2004). Cleavage of double stranded plasmid DNA by lanthanide complexes. J. Chromatogr. B Analyt. Technol. Biomed. Life Sci..

[17] Tenan M.R., Nicolle A., Moralli D., Verbouwe E., Jankowska J.D., Durin M.A., Green C.M., Mandriota S.J., Sappino A.P. (2021). Aluminum Enters Mammalian Cells and Destabilizes Chromosome Structure and Number. Int. J. Mol. Sci..

[18] Zhang S., Hao S., Qiu Z., Wang Y., Zhao Y., Li Y., Gao W., Wu Y., Liu C., Xu X. (2019). Cadmium disrupts the DNA damage response by destabilizing RNF168. Food Chem. Toxicol..

[19] Shah A.J., Lakkad B.C., Rao M.V. (2016). Genotoxicity in lead treated human lymphocytes evaluated by micronucleus and comet assays. Indian J. Exp. Biol..

[20] DeLoughery Z., Luczak M.W., Ortega-Atienza S., Zhitkovich A. (2015). DNA double-strand breaks by Cr(VI) are targeted to euchromatin and cause ATR-dependent phosphorylation of histone H2AX and its ubiquitination. Toxicol. Sci. Off. J. Soc. Toxicol..

[21] Pottier G., Viau M., Ricoul M., Shim G., Bellamy M., Cuceu C., Hempel W.M., Sabatier L. (2013). Lead Exposure Induces Telomere Instability in Human Cells. PLoS ONE.

[22] Ayene I.S., Koch C.J., Krisch R.E. (2007). DNA strand breakage by bivalent metal ions and ionizing radiation. Int. J. Radiat. Biol..

[23] Rothkamm K., Lobrich M. (2003). Evidence for a lack of DNA double-strand break repair in human cells exposed to very low x-ray doses. Proc. Natl. Acad. Sci. USA.

[24] Granzotto A., Benadjaoud M.A., Vogin G., Devic C., Ferlazzo M.L., Bodgi L., Pereira S., Sonzogni L., Forcheron F., Viau M. (2016). Influence of Nucleoshuttling of the ATM Protein in the Healthy Tissues Response to Radiation Therapy: Toward a Molecular Classification of Human Radiosensitivity. Int. J. Radiat. Oncol. Biol. Phys..

[25] Belkacemi Y., Colson-Durand L., Granzotto A., Husheng S., To N.H., Majdoul S., Guet S., Herve M.L., Fonteneau G., Diana C. (2016). The Henri Mondor Procedure of Morbidity and Mortality Review Meetings: Prospective Registration of Clinical, Dosimetric, and Individual Radiosensitivity Data of Patients With Severe Radiation Toxicity. Int. J. Radiat. Oncol. Biol. Phys..

[26] Pereira S., Bodgi L., Duclos M., Canet A., Ferlazzo M.L., Devic C., Granzotto A., Deneuve S., Vogin G., Foray N. (2018). Fast and binary assay for predicting radiosensitivity based on the nucleoshuttling of ATM protein: Development, validation and performances. Int. J. Radiat. Oncol. Biol. Phys..

[27] Vogin G., Bastogne T., Bodgi L., Gillet-Daubin J., Canet A., Pereira S., Foray N. (2018). The Phosphorylated ATM Immunofluorescence Assay: A High-performance Radiosensitivity Assay to Predict Postradiation Therapy Overreactions. Int. J. Radiat. Oncol. Biol. Phys..

[28] Maalouf M., Granzotto A., Devic C., Bodgi L., Ferlazzo M., Peaucelle C., Bajard M., Giraud J.Y., Balosso J., Herault J. (2019). Influence of Linear Energy Transfer on the Nucleo-shuttling of the ATM Protein: A Novel Biological Interpretation Relevant for Particles and Radiation. Int. J. Radiat. Oncol. Biol. Phys..

[29] Berthel E., Ferlazzo M., Devic C., Bourguignon M., Foray N. (2019). What does the History of Research on the Repair of DNA Double-Strand Breaks Tell Us?—A Comprehensive Review of Human Radiosensitivity. Int. J. Mol. Sci..

[30] Berthel E., Foray N., Ferlazzo M.L. (2019). The Nucleoshuttling of the ATM Protein: A Unified Model to Describe the Individual Response to High- and Low-Dose of Radiation?. Cancers.

[31] Bodgi L., Foray N. (2016). The nucleo-shuttling of the ATM protein as a basis for a novel theory of radiation response: Resolution of the linear-quadratic model. Int. J. Radiat. Biol..

[32] Foray N., Fertil B., Alsbeih M.G., Badie C., Chavaudra N., Iliakis G., Malaise E.P. (1996). Dose-rate effect on radiation-induced DNA double-strand breaks in the human fibroblast HF19 cell line. Int. J. Radiat. Biol..

[33] Foray N., Priestley A., Alsbeih G., Badie C., Capulas E.P., Arlett C.F., Malaise E.P. (1997). Hypersensitivity of ataxia telangiectasia fibroblasts to ionizing radiation is associated with a repair deficiency of DNA double-strand breaks. Int. J. Radiat. Biol..

[34] Gastaldo J., Bencokova Z., Massart C., Joubert A., Balosso J., Charvet A.M., Foray N. (2011). Specific molecular and cellular events induced by irradiated X-ray photoactivatable drugs raise the problem of co-toxicities: Particular consequences for anti-cancer synchrotron therapy. J. Synchrotron Radiat..

[35] Varela I., Pereira S., Ugalde A.P., Navarro C.L., Suarez M.F., Cau P., Cadinanos J., Osorio F.G., Foray N., Cobo J. (2008). Combined treatment with statins and aminobisphosphonates extends longevity in a mouse model of human premature aging. Nat. Med..

[36] Foray N., Marot D., Gabriel A., Randrianarison V., Carr A.M., Perricaudet M., Ashworth A., Jeggo P. (2003). A subset of ATM- and ATR-dependent phosphorylation events requires the BRCA1 protein. EMBO J..

[37] Ferlazzo M., Berthel E., Granzotto A., Devic C., Sonzogni L., Bachelet J.T., Pereira S., Bourguignon M., Sarasin A., Mezzina M. (2019). Some mutations in the xeroderma pigmentosum D gene may lead to moderate but significant radiosensitivity associated with a delayed radiation-induced ATM nuclear localization. Int. J. Radiat. Biol..

[38] Colin C., Devic C., Noël A., Rabilloud M., Zabot M.-T., Pinet-Isaac S., Giraud S., Riche B., Valette P.-J., Rodriguez-Lafrasse C. (2011). DNA double-strand breaks induced by mammographic screening procedures in human mammary epithelial cells. Int. J. Radiat. Biol..

[39] Bodgi L., Granzotto A., Devic C., Vogin G., Lesne A., Bottollier-Depois J.F., Victor J.M., Maalouf M., Fares G., Foray N. (2013). A single formula to describe radiation-induced protein relocalization: Towards a mathematical definition of individual radiosensitivity. Int. J. Theor. Biol..

[40] Grote S.J., Joshi G.P., Revell S.H., Shaw C.A. (1981). Observations of radiation-induced chromosome fragment loss in live mammalian cells in culture, and its effect on colony-forming ability. Int. J. Radiat. Biol. Relat. Stud. Phys. Chem. Med..

[41] Nascarella M.A., Stoffolano Jr J.G., Stanek 3rd E.J., Kostecki P.T., Calabrese E.J. (2003). Hormesis and stage specific toxicity induced by cadmium in an insect model, the queen blowfly, Phormia regina Meig. Environ. Pollut..

[42] Bleavins K., Perone P., Naik M., Rehman M., Aslam M.N., Dame M.K., Meshinchi S., Bhagavathula N., Varani J. (2012). Stimulation of fibroblast proliferation by insoluble gadolinium salts. Biol. Trace Elem. Res..

[43] Philips N., Hwang H., Chauhan S., Leonardi D., Gonzalez S. (2010). Stimulation of cell proliferation and expression of matrixmetalloproteinase-1 and interluekin-8 genes in dermal fibroblasts by copper. Connect Tissue Res..

[44] Beyersmann D., Haase H. (2001). Functions of zinc in signaling, proliferation and differentiation of mammalian cells. Biometals.

[45] Wagner S., Hussain M.Z., Hunt T.K., Bacic B., Becker H.D. (2004). Stimulation of fibroblast proliferation by lactate-mediated oxidants. Wound Repair Regen..

[46] Ferlazzo M.L., Bach-Tobdji M.K.E., Djerad A., Sonzogni L., Burlet S.F., Devic C., Granzotto A., Bodgi L., Djeffal-Kerrar A., Foray N. (2017). Radiobiological characterization of tuberous sclerosis: A delay in the nucleo-shuttling of ATM may be responsible for radiosensitivity. Mol. Neurobiol..

[47] Ferlazzo M.L., Sonzogni L., Granzotto A., Bodgi L., Lartin O., Devic C., Vogin G., Pereira S., Foray N. (2014). Mutations of the Huntington’s Disease Protein Impact on the ATM-Dependent Signaling and Repair Pathways of the Radiation-Induced DNA Double-Strand Breaks: Corrective Effect of Statins and Bisphosphonates. Mol. Neurobiol..

[48] Varela I., Cadinanos J., Pendas A.M., Gutierrez-Fernandez A., Folgueras A.R., Sanchez L.M., Zhou Z., Rodriguez F.J., Stewart C.L., Vega J.A. (2005). Accelerated ageing in mice deficient in Zmpste24 protease is linked to p53 signalling activation. Nature.

[49] Renier W., Joubert A., Bencokova Z., Gastaldo J., Massart C., Foray N. (2007). Consequences of the bleed-through phenomenon in immunofluorescence of proteins forming radiation-induced nuclear foci. Int. J. Radiat. Biol..

[50] El-Nachef L., Al-Choboq J., Restier-Verlet J., Granzotto A., Berthel E., Sonzogni L., Ferlazzo M.L., Bouchet A., Leblond P., Combemale P. (2021). Human Radiosensitivity and Radiosusceptibility: What Are the Differences?. Int. J. Mol. Sci..

[51] Joiner M.C., Marples B., Lambin P., Short S.C., Turesson I. (2001). Low-dose hypersensitivity: Current status and possible mechanisms. Int. J. Radiat. Oncol. Biol. Phys..

[52] Calabrese E.J. (2008). Hormesis is central to toxicology, pharmacology and risk assessment. Hum. Exp. Toxicol..

[53] Calabrese E.J. (2014). Hormesis: A fundamental concept in biology. Microb. Cell.

[54] Devic C., Ferlazzo M.L., Foray N. (2018). Influence of Individual Radiosensitivity on the Adaptive Response Phenomenon: Toward a Mechanistic Explanation Based on the Nucleo-Shuttling of ATM Protein. Dose-Response Publ. Int. Hormesis Soc..

[55] Granzotto A., Bencokova Z., Vogin G., Devic C., Joubert A., Balosso J., Foray N., Abujamra A.L. (2011). DNA double-strand breaks repair and signaling of human gliomas and normal brain cells in response to radiation: Po-tential impact of the ATM- and BRCA1-dependent pathways. Brain Tumors/Book 3.

[56] Hartwig A. (1995). Current aspects in metal genotoxicity. Biometals.

[57] Lay P.A., Levina A. (1998). Activation of Molecular Oxygen during the Reactions of Chromium(VI/V/IV) with Biological Reductants: Implications for Chromium-Induced Genotoxicities. J. Am. Chem. Soc..

[58] Levina A., Codd R., Dillon C.T., Lay P.A., Karlin K.D. (2003). Chromium in Biology. Toxicology and Nutritional Aspects Progress in Inorganic Chemistry.

[59] Averbeck D., Candeias S., Chandna S., Foray N., Friedl A.A., Haghdoost S., Jeggo P.A., Lumniczky K., Paris F., Quintens R. (2020). Establishing mechanisms affecting the individual response to ionizing radiation. Int. J. Radiat. Biol..

[60] Misono M., Ochini E., Saito Y., Yoneda Y. (1967). A new dual parameter scale for the strength of Lewis acids and bases with the evaluation of their softness. J. Inorg. Nucl. Chem..

[61] Pearson R.G. (1968). Hard and Soft acids and bases, Part I. J. Chem. Educ..

[62] WHO (2004). Guidelines for Drinking-Water Quality.

[63] Paull T.T. (2015). Mechanisms of ATM Activation. Annu. Rev. Biochem..

[64] Pagacova E., Stefancikova L., Schmidt-Kaler F., Hildenbrand G., Vicar T., Depes D., Lee J.H., Bestvater F., Lacombe S., Porcel E. (2019). Challenges and Contradictions of Metal Nano-Particle Applications for Radio-Sensitivity Enhancement in Cancer Therapy. Int. J. Mol. Sci..

