# Supplementary material for: DNA Double-Strand Breaks Induced in Human Cells by Twelve Metallic Species: Quantitative Inter-Comparisons and Influence of the ATM Protein
Source: Biomolecules. 2021 Oct 5;11(10):1462. doi: 10.3390/biom11101462 (PMC8533583; doi:10.3390/biom11101462)
Supplement: Supplementary file 1 [file biomolecules-11-01462-s001.zip › biomolecules-1361012-supplementary.pdf]

DNA double-strand breaks induced in human cells by twelve metallic species: quantitative inter-comparisons and influence of the ATM protein

Supplementary Data

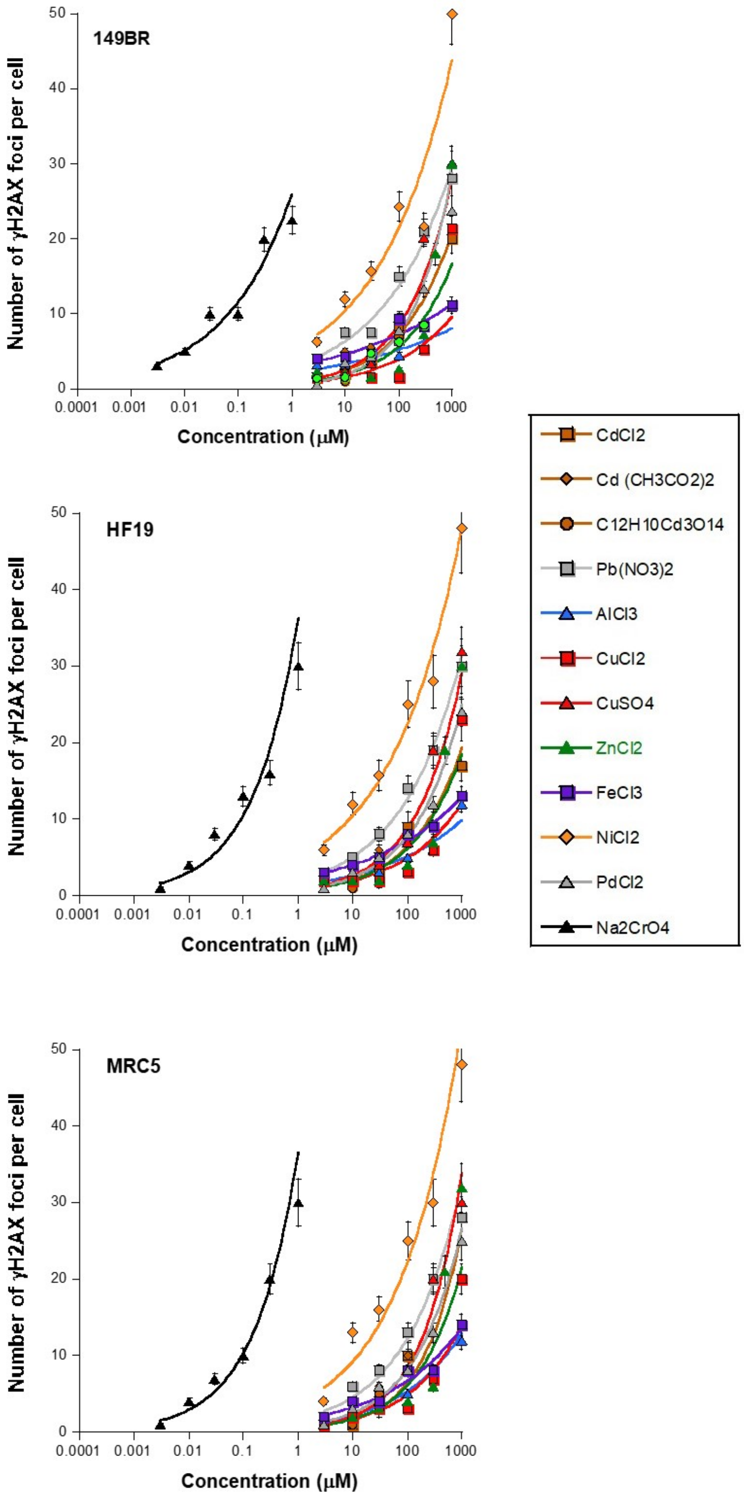

Figure S1: Unrepaired DSB after metal exposure in different fibroblast cell lines. Number of γH2AX foci per cell in the human untransformed radioresistant 149BR, HF19 and MRC5 cells after incubation for 24 h with the indicated concentration of metal species plotted. For 149BR data, each plot represents the mean ± standard error (SEM) of at least three replicates (reproduction of the Fig. 1B shown in the article). For HF19 and MRC5 data, each plot represents the mean ± standard error (SEM) of at least two replicates.

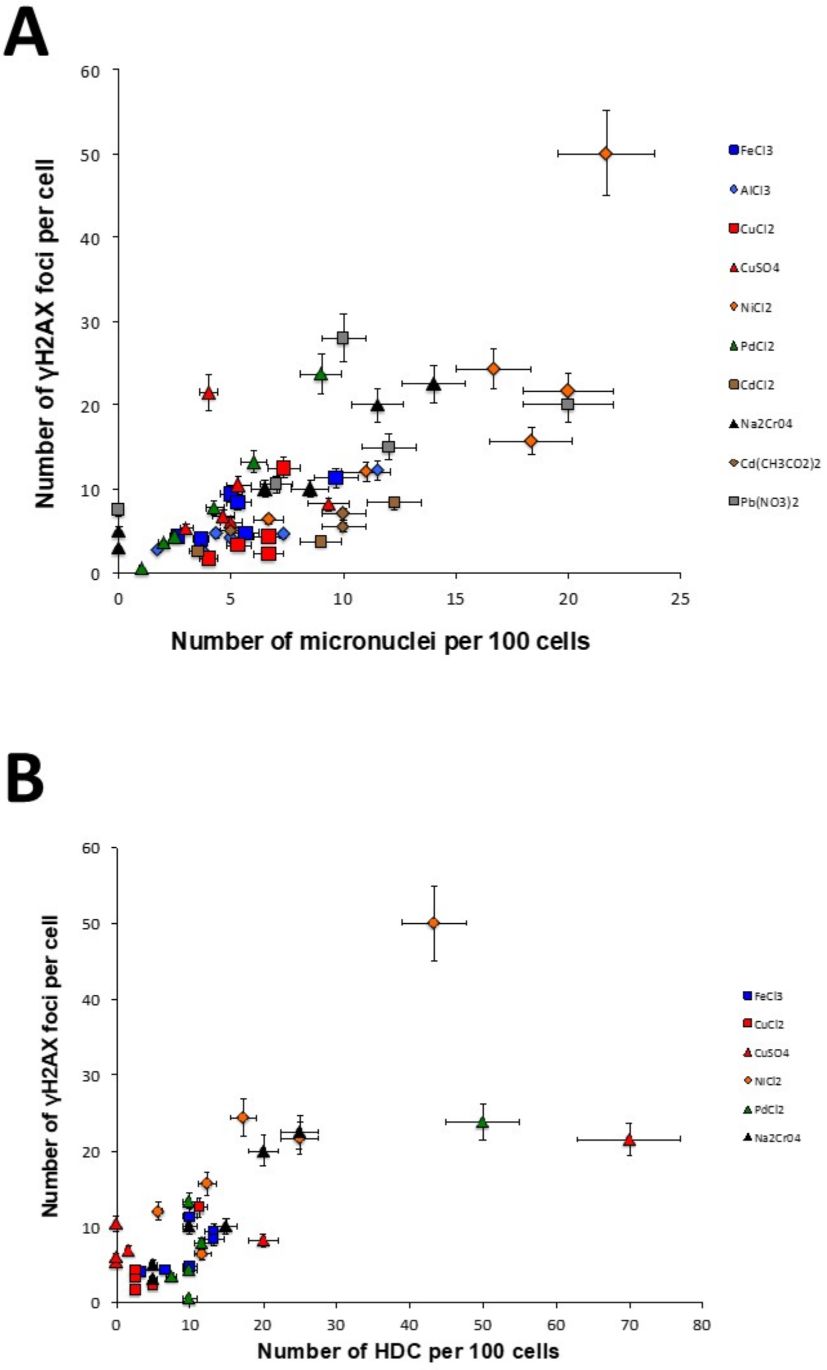

Figure S2: Residual micronuclei and highly damaged cells after metal contamination. A. The γH2AX data shown in Figure 1 were plotted against the corresponding micronuclei ones shown in Figure 2. Linear data fitting provided the formula $y = 1.2x \pm 0.3$ ($r=0.8$; $p=0.04$). **B.** The γH2AX data shown in Figure 1 were plotted against the corresponding HDC ones shown in Figure 2B. Linear data fitting provided the formula $y=1.0x \pm 0.3$ ($r=0.85$; $p=0.04$).

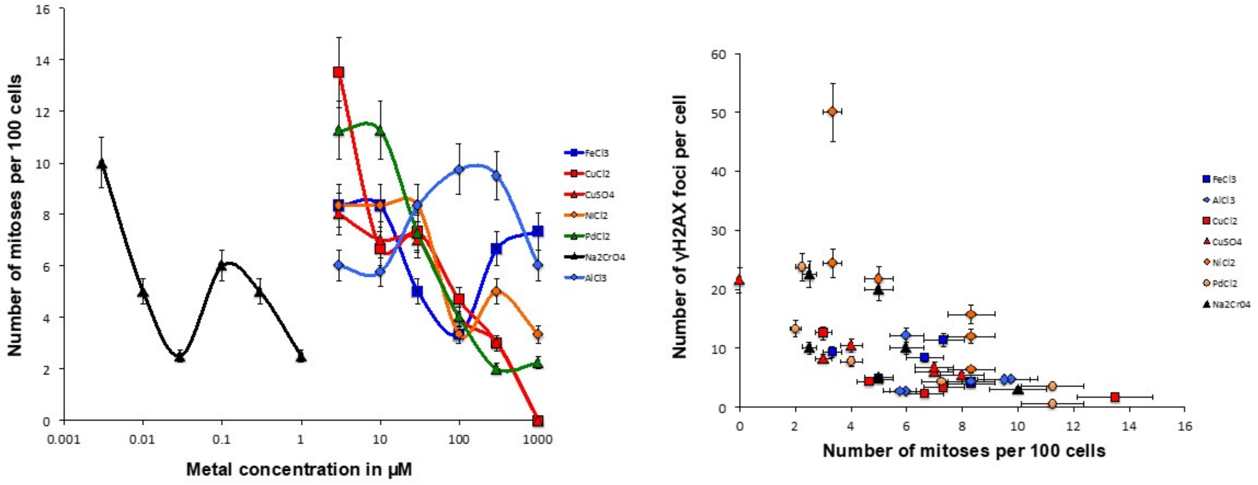

Figure S3: Mitoses and contamination with metal. Left. Number of mitoses per 100 cells in the human untransformed radioresistant 149BR cells after incubation for 24 h with the indicated concentration of metal species. Each plot represents the mean ± standard error (SEM) of at least three replicates. Right. The γH2AX data shown in Figure 1 were plotted against the corresponding the mitoses data shown in the right panel.

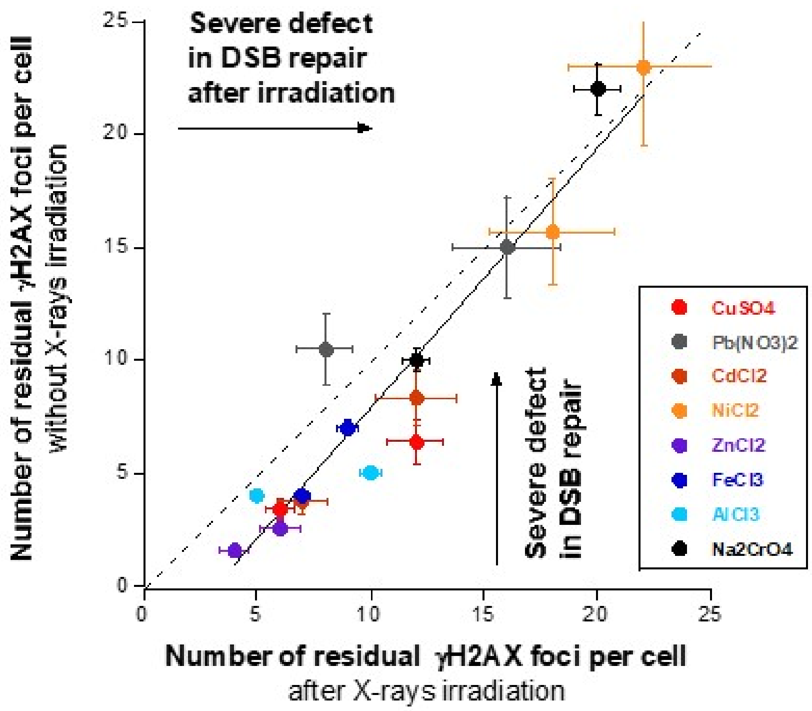

Figure S4: Correlation between γH2AX data obtained after 24 h contamination with metal followed by irradiation and γH2AX data obtained without contamination with metal. The number of γH2AX foci assessed after 24 h contamination with metal shown in Figure 1 were plotted against the number of of γH2AX foci assessed after 24 h contamination with metal followed by an irradiation followed itself by 24 h repair time. The dotted line corresponds to a one to one correlation. The solid line is the result of a data fitting to a linear function.

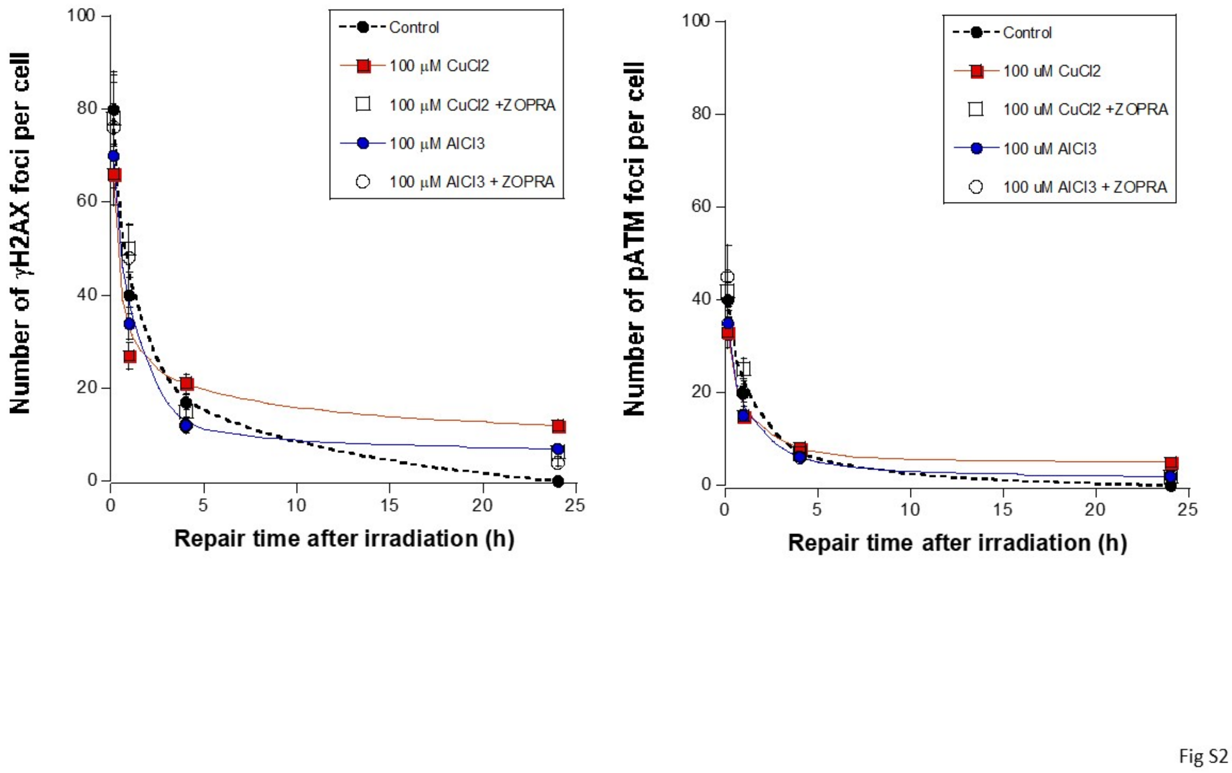

Figure S5: Effect of ZoPra treatment and contamination with metal: Number of γH2AX (left) and pATM (right) foci as a function of repair time post-irradiation in the human untransformed radioresistant 149BR incubated for 24 h with the indicated concentration of metal species and treated or not by the ZoPra treatment. It is noteworthy that ZoPra treatment did not affect the foci kinetics of controls (data not shown). Each plot represents the mean ± standard error (SEM) of at least two replicates.

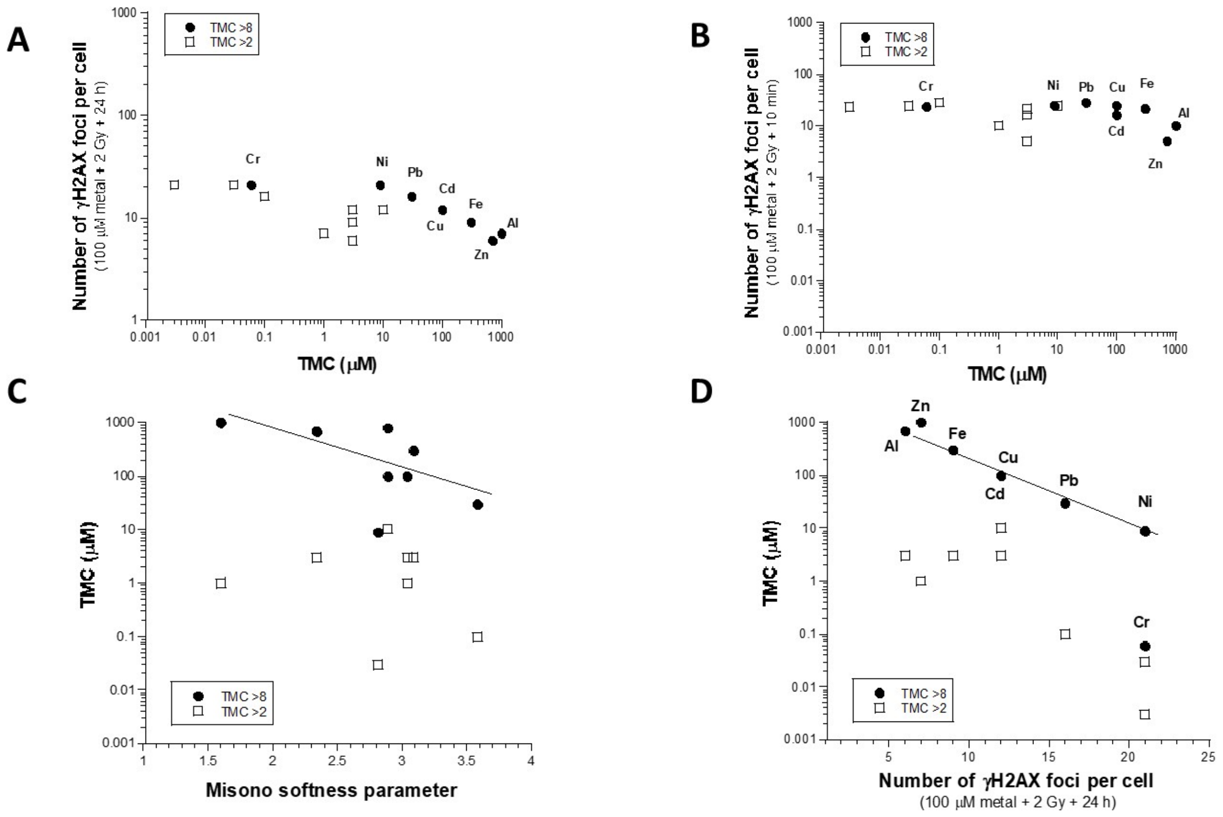

Figure S6: Relationships between the TMC, the number of γH2AX foci and the Misono softness parameters. A and B: Relationships between the TMC values and the number of γH2AX foci assessed 24 h and 10 min post-irradiation. All these values are shown in Table 2**. C and D. :** Relationships between the TMC values and the Misono softness parameter (C) or the number of γH2AX foci assessed 24 h post-irradiation (D). All these values are shown in Table 2**.** Continuous line reflects the data fitting with an exponential law : C : $y=25.2\ e^{-1.7x}$; r=0.75; D: $y=5.43\ e^{-0.31x}$; r=0.90

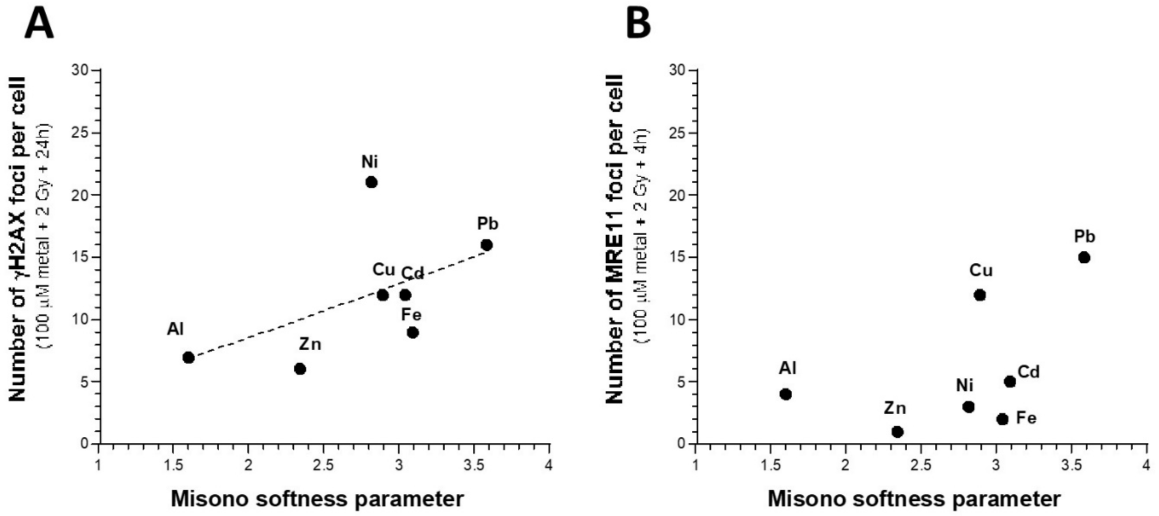

Figure S7: Relationships between the number of γH2AX and MRE11 foci and the Misono softness parameter. Relationships between the number of γH2AX foci assessed 24 h post-irradiation (A), the number of MRE11 foci assessed 4 h post-irradiation (B) and the Misono softness parameter. All these values are shown in Table 2, Figure 5 C and D**.** Dotted line reflects the data fitting with a linear law: A : $y=4.3\ x$; $r=0.54$;
